# Health-Related Quality of Life across Recent Pediatric Obesity Classification Recommendations

**DOI:** 10.3390/children8040303

**Published:** 2021-04-15

**Authors:** William R. Black, Kelsey B. Borner, Marshall T. Beauchamp, Ann M. Davis, Meredith L. Dreyer Gillette, Brooke Sweeney, Sarah E. Hampl

**Affiliations:** 1 Center for Children’s Healthy Lifestyles & Nutrition, Kansas City, MO 64108, USA; adavis6@kumc.edu (A.M.D.); mldreyer@cmh.edu (M.L.D.G.); brsweeney@cmh.edu (B.S.); shampl@cmh.edu (S.E.H.); 2 Department of Pediatrics, The University of Kansas Medical Center, Kansas City, KS 66160, USA; 3 Department of Psychology and Behavioral Health, Children’s National Hospital, Washington, DC 20010, USA; kborner@childrensnational.org; 4 Department of Psychology, University of Missouri–Kansas City, Kansas City, MO 6110, USA; marshallbeauchamp@gmail.com; 5 Department of Pediatrics, Children’s Mercy Kansas City, Kansas City, MO 64108, USA

**Keywords:** health-related quality of life, child obesity, obesity category, obesity class, BMI percentage

## Abstract

Extreme body mass index (BMI) values (i.e., above the 97th and below the 3rd percentiles) are inaccurately represented on the Centers for Disease Control and Prevention’s growth curves, which may limit the utility of BMI percentile and BMI z-score for capturing changes in clinical outcomes for patients at extreme weights. Modeling child obesity severity based upon the percentage of BMI in excess of the 95th percentile (BMI95pct) has been proposed as an improved metric to better capture variability in weight at extreme ends of growth curves, which may improve our understanding of relationships between weight status and changes in clinical outcomes. However, few studies have evaluated whether the use of BMI95pct would refine our understanding of differences in clinical psychosocial constructs compared to previous methods for categorization. This cross-sectional study evaluated child obesity severity based on BMI95pct to examine potential group differences in a validated, obesity-specific measure of Health-Related Quality of Life (HRQoL). Four hundred and sixty-five children with obesity completed Sizing Me Up, a self-report measure of HRQoL. Children were classified into categories based on BMI95pct (i.e., class I: ≥100% and <120%; class II: ≥120% and <140%; class III: ≥140%). The results indicate that children with class III obesity reported lower HRQoL than children with class I and class II obesity; however, there were no differences between Class II and Class I. In much of the previous literature, children with class II and class III obesity are often combined under the category “Severe Obesity” based upon BMI above the 99th percentile. This study suggests that grouping children from various classes together would neglect to capture critical differences in HRQoL. Future research including children with severe obesity should consider obesity classes to best account for functioning and clinical outcomes.

## 1. Introduction

Pediatric obesity affects one sixth of all U.S. children, and 5.8% of these children are considered to have severe obesity [1,2]. Obesity is defined by a Body-Mass-Index (BMI) ≥ 95th percentile for age and gender, while severe obesity classifies those with BMIs ≥ 99th percentile on the 2000 Centers for Disease Control and Prevention (CDC) growth curves [2]. These curves were developed using statistical smoothing and normalizing procedures that allowed for the calculation of percentiles between the 3rd and 97th percentile and data outside of these ranges were not modeled as part of these curves. Subsequently, while BMI percentile and standardized BMI scores (BMIz or BMI z-score) are regularly used as objective measures of treatment outcomes and for providing obesity education to patients and families [3,4], these original curves most accurately reflect patients with BMI scores between the 3rd and 97th percentiles.

These curves have also been used to plot and model children at higher levels of obesity, despite significant limitations. Per Freedman and colleagues [5,6], there are several additional reasons why representing extreme BMI percentile values on the CDC curves is problematic. First, at extreme BMI values, BMIz scores become compressed, with multiple BMI values equating to a similar z-score. Thus, individuals with very similar z-scores may have vastly different clinical profiles, meaning that at very high BMIz scores, weight loss may not be represented in BMIz change scores. Second, there exists a theoretical maximum value for BMIz that can be obtained for a given sex and age [7], and there is large variability regarding these values in children with severe obesity [8]. Consequently, existing models are a poor fit for individuals with extreme BMI values (e.g., above the 97th percentile and below the 3rd percentile) [2,9,10] and their use has been cautioned against when representing the weight status of those outside this range [9,11].

As a solution, several researchers have recommended that pediatric weight status in the extreme upper values is better represented as percentage of the 95th BMI percentile (BMI95pct) [2,9,10]. Instead of comparing a child’s BMI to the normative population, as is done with BMI percentile or BMI z-score, BMI95pct evaluates a child’s BMI as compared to the expected BMI at the 95th percentile for the child’s age, height, weight, and gender. This metric significantly expands the range of measurement for severe obesity and allows for better differentiation of weight status for children with severe obesity. For example, a child whose BMI95pct score is equal to 100 has a BMI that is on the 95th percentile, whereas a child whose BMI95pct score is equal to 120 has a BMI that is 20% higher than their expected BMI score at the 95th percentile—effectively measuring obesity according to a new reference point (i.e., the 95th percentile instead of the normal population).

New obesity severity classification systems have been proposed utilizing BMI95pct [12,13,14,15]. In this new system, class I obesity is defined as BMI95pct ≥100% and <120%, class II obesity BMI95pct ≥120% and <140%, and class III obesity is BMI95pct ≥140%. Using this classification system, the National Health and Nutrition Examination Survey (NHANES) data from 1999 through 2016 revealed that 31.2% of children between 2 and 19 years old had overweight, while 16.4% presented with class I obesity, 5.1% with class II obesity, and 1.5% with class III obesity [13].

This classification system is also being increasingly used to evaluate medical outcomes in children with severe obesity. Studies have found that children with class II obesity demonstrated greater risk for abnormal levels of HDL cholesterol, systolic blood pressure, and glucose, and those with class III obesity had significantly worse levels of triglycerides, diastolic blood pressure, and glycated hemoglobin compared to children with class II obesity [12,16]. Such data support the use and inclusion of class II and class III obesity for predicting some clinical metrics, such as metabolic risk in children, and demonstrate that the two classes have different health risk factors and complications. The 2007 American Academy of Pediatrics expert recommendations for pediatric obesity do not differentiate between classes of obesity [17], while more recent recommendations, such as those from the Endocrine Society [18], do advocate for a classification approach. This evolution in classification using Class I-III obesity may allow for increased discrimination of clinical differences among patients with a BMI greater than the 95th percentile [12,13]. However, further evidence for the clinical utility of this classification system is needed, particularly among other health-related and psychosocial outcomes.

### The Current Study

Health-related quality of life (HRQoL) is a well-established and meaningful multidimensional indicator of patient well-being and functioning and is utilized in pediatric obesity literature [19,20]. Poorer HRQoL has been well-documented amongst children with higher weight status [20,21,22], and has also been shown to differ across racial and ethnic groups on some subscales [23]. While previous literature has demonstrated the unique association between higher classes of severe obesity and impaired HRQoL among adults [24,25], to the best of our knowledge, no study has evaluated psychosocial outcomes by obesity class according to BMI95pct classification in a pediatric sample. The current cross-sectional study explores the clinical utility of BMI severity classification according to BMI95pct by examining relationships and group differences in one measure of psychosocial functioning (i.e., Health-Related Quality of Life). It is hypothesized that children with class III obesity will exhibit significantly poorer HRQoL than children with class I and class II, and that children with class II obesity will also exhibit significantly poorer HRQoL than children with class I obesity.

## 2. Materials and Methods

### 2.1. Participants and Procedure

Participants were children with obesity and their families seeking treatment in one of two family-based behavioral pediatric weight-management programs. Inclusion criteria for both programs included having a BMI over the 85th percentile for age and gender and having an English- or Spanish-speaking parent or caregiver who could attend the program with the child. Exclusion criteria included diagnoses that would preclude participation in a group (e.g., severe autism). Additional information about the format of the treatment programs is available elsewhere [26,27]. The present analysis included children between the ages of 5 and 13 years with BMIs above the 95th percentile from either program. Weight, height, and self-reported HRQoL were taken at baseline for each program, at a separate visit from their initial study visit. All baseline visits were conducted within 2 weeks of the first program visit. This study was reviewed and approved by the Institutional Review Boards of both study sites, the University of Kansas Medical Center’s (#10351) and Children’s Mercy Hospitals’ (#11120473).

### 2.2. Measures and Materials

#### 2.2.1. Demographics and Anthropometry

Participant age, sex, and race were obtained from the medical record. Height was measured to the closest 0.1 cm using a stadiometer and weight was measured to the closest 0.1 kg using a digital scale. Height, weight, age, and sex were used in conjunction with Statistical Analysis System (SAS) code available through the CDC to calculate BMI95pct [28].

#### 2.2.2. Health Related Quality of Life (HRQoL)—Sizing Me Up

HRQoL was assessed via Sizing Me Up (SMU), a 22-item obesity-specific self-report measure designed to evaluate HRQoL in children between 5 and 13 years of age [22]. SMU consists of a Total HRQoL score and several subscales (i.e., Emotional Functioning, Physical Functioning, Social Avoidance, Positive Social Attributes, and Teasing/Marginalization) which capture children’s perceptions of how their size makes them feel, inhibits their ability to engage in physical activities, inhibits their ability to engage in social activities, relates to positive traits, and makes them feel excluded or teased by peers. For younger children, and as needed, SMU was administered by a research assistant by either reading survey questions to the child or by clarifying terms and word meaning. SMU has good internal consistency among the different scales (*α* = 0.68–0.85, *α_Total_* = 0.82), test–retest reliability (*r* = 0.35–0.74, *r*_Total_ = 0.78), and convergent validity with other similar HRQoL measures (e.g., PedsQL; *r* = 0.35–0.65, *r*_Total_ = 0.38–0.55). Reliability of the SMU in this study ranged from *α* = 0.775–0.857 across the three obesity classes. Scores range from 0–100 and higher scores indicate better obesity-specific HRQoL.

#### 2.2.3. Statistical Analysis

Data were analyzed using the Statistical Package for the Social Sciences (SPSS) version 18.0 [29] and SAS University Edition. [28] Descriptive statistics were calculated for all variables, and correlations (i.e., Pearson’s and point-biserial) and regression coefficients were calculated to evaluate covariability between age, sex and HRQoL. Sex was dummy coded with male = 0. Pearson’s correlations were also conducted between HRQoL and BMI95pct to evaluate preliminary relationships between quality of life and weight status. An Analysis of Covariance (ANCOVA), controlling for sex and age, compared HRQoL between the three subgroups of children (i.e., class I, class II, and class III obesity). To evaluate the appropriateness of ANCOVA (i.e., model diagnostics), Levene’s Test was used to evaluate error variance across the obesity groups, and White’s Test of Heteroskedasticity was used to evaluate differences in error variances across groups. Fisher’s Least Significant Difference was utilized to evaluate post hoc pairwise comparisons. Cohen’s *d* was calculated to evaluate effect size and is interpreted (e.g., small [*d* = 0.1], medium [*d* = 0.3], large [*d* = 0.5]) according to recommendations introduced by Cohen [30]. Statistical significance is set at α = 0.05 (i.e., *p* < 0.05). No individuals were omitted from the primary analyses due to missing data; however, four participants did not identify race or ethnicity and were omitted from ethnicity-based analyses. The Benjamini–Hochberg procedure was used to account for multiple test corrections within sets of analyses, with a conservative false discovery rate of 0.05 [31]. A post hoc power analysis was also conducted using G*Power 3.1 [32]. For two-tailed correlational analyses, with an estimated power of β = 0.8 and error probably of α = 0.05. For whole sample (*n* = 465) correlations, this study is powered to detect correlations above *r* = 0.13, and correlations of *r* = 0.22 or greater for each obesity class. Post hoc power analyses for a 3-group ANCOVA with 2 covariates and standard power and error assumptions (β = 0.8, α = 0.05) demonstrates that with this study’s sample size, medium effect sizes can be detected (*d* = 0.35).

## 3. Results

### 3.1. Demographic Data and HRQoL

Of the children and families who enrolled in either of the treatment programs (*n* = 557), 465 met inclusion criteria and had complete data (i.e., demographic, anthropometric, and HRQoL) (see Table 1 for Demographics). Participants had a mean age of 10.84 years old (*SD* = 1.89), and 53.8% were female. Child race was reported as: 23% White, 32% Black/African American, and 43% Hispanic/Latino. On SMU, children reported a Total HRQoL score of 68.25 (*SD* = 14.80) (see Table 2 for subscale scores). Average BMI percentile was 98.48 (*SD* = 0.96) and did not significantly differ between the two treatment sites (*p* = 0.131). Participants were well balanced across obesity classes.

### 3.2. Demographic Covariates and HRQoL

To determine whether participant age and sex needed to be included as covariates for other analyses, child correlations between age and sex and individual HRQoL subscales were conducted (see Table 1). Several subscales, including Total HRQoL were significantly (i.e., *p* < 0.05) correlated with age and sex. Subsequently, age and sex were included as covariates in the ANCOVA.

### 3.3. Obesity Class Comparisons

BMI95pct was significantly associated with Total HRQoL (*r* = −0.19, *p* < 0.001), and was significantly associated with Emotional Functioning (*r* = −0.15, *p* < 0.001), Physical Functioning (*r* = −0.23, *p* < 0.001), Social Avoidance (*r* = −0.15, *p* < 0.001), and Teasing/Marginalization (*r* = −0.26, *p* < 0.001). Obesity classes were then compared using a one-way ANCOVA and were found to significantly differ from one another, *F*(2, 460) = 13.46, *p* < 0.001. Post hoc pairwise comparisons found that children with class III obesity reported lower Total HRQoL than children with class I obesity (*p* < 0.001) and children with class II obesity (*p* < 0.001), and that this group difference was a medium effect size (*d* = 0.50). All of these reported analyses were significant after family-wise error correction. However, total and subscale scores for children with class I and class II obesity did not differ from each other. Estimated marginal means and comparisons for Total HRQoL and subscale scores are presented in Table 3.

### 3.4. Obesity Class Differences across Ethnicity Groups

BMI95pct significantly differed across the three ethnicity groups, *F*(2, 453) = 13.216, *p* < 0.001. Tukey’s post hoc pairwise comparisons found that children who identified as Black or African American (*M* = 142.22, *SE* = 1.81, 95% CI: 138.67, 145.77) had significantly greater BMI95pct than Hispanic/Latino children (*M* = 125.27, *SE* = 1.58, 95% CI: 122.16, 128.37) (*p* = 0.003), but not White/Caucasian children (*M* = 135.82, *SE* = 2.09, 95% CI: 131.72, 139.91) (*p* = 0.069). White/Caucasian children did not significant differ from Black/African American children on mean BMI95pct (*p* = 0.351). Additionally, a Chi-Square test of independence was conducted to examine proportional differences in race/ethnicity across the three obesity groups, and the distribution of race/ethnicity was not equal across the three obesity groups, χ^2^ (4) = 37.50, *p* < 0.001. Within-group percentages across obesity groups are shown in Table 1. While White/Caucasian children are fairly evenly distributed across obesity groups, Black/African American children are most likely to present with Class III obesity (47.3%) and least likely to present with Class I (18.9%). Conversely, Hispanic/Latino children were most likely to present with Class I obesity and least likely to present with Class III obesity (19.6%).

### 3.5. HRQoL Differences between Ethnicity Groups and Obesity Classes

Total HRQoL was compared across race/ethnicity groups via ANCOVA and found to differ significantly across groups, *F*(2, 453) = 3.584, *p = 0*.007. Post hoc analyses (see Table 4) found Hispanic/Latino children presented with poorer Total HRQoL than White/Caucasian children (*p = 0*.049). However, when analyzed within each obesity class, no statistically significant differences in Total HRQoL were found (*p* > 0.05).

## 4. Discussion

This study was the first to assess the relationship between obesity class and HRQoL in a pediatric sample, and the first, to our knowledge, that evaluated a psychosocial outcome by childhood obesity class. This study found that severe obesity class differences are present in this important psychosocial outcome. This aligns with previous research reporting a relationship between obesity class and medical outcomes in pediatric patients. Not only has the use of BMI95pct been shown to be a statistically better fit for extreme obesity values [10,11], the results of this study align with clinical studies that have found BMI95pct to be more predictive of medical complications and risk factors associated with pediatric obesity than previous classifications [4,13,15], providing further support for the use of a severe obesity classification system among pediatric populations.

Surprisingly, study hypotheses were only partially supported. Children with class III obesity reported significantly lower HRQoL than those with class II and class I obesity. These group differences were not small—the medium effect sizes suggest that group differences observed in this study were not an artifact of a large sample size and may represent clinically meaningful effects. However, in contrast to our expectations, and of particular interest, HRQoL total and subscale scores for children with class II obesity did not differ from those for children with class I obesity. Thus, while children with class III obesity were distinct from their peers, children with class II obesity did not report significantly greater impairment than those with class I obesity.

The unique effect of class III obesity on HRQoL and other psychosocial outcomes has been seen in the adult literature. In a meta-analysis of eight studies, Ul-Haq and colleagues (2013) demonstrated that, while reductions in physical HRQoL evidenced a dose relationship between the three severe obesity classes, emotional HRQoL was significantly reduced among adults with class III obesity, but not significantly different among those with class I or class II obesity. Further, significant differences in sleep quality and depression have been shown among female cancer survivors with class III obesity [33], which suggests that the association between the upper levels of severe obesity and poorer HRQoL may be related to other sequelae of obesity. Future research is needed to further elucidate the factors that may contribute to poorer HRQoL among children with class III obesity, such as sleep disturbances, depressed mood, and decreased physical activity.

This study demonstrates that impairment in psychosocial functioning is also not uniform across children with severe obesity and that children with the greatest amount of excess body weight (class III) self-report subjectively greater impairment compared to children with both class I obesity and class II severe obesity. The qualitative experience of children with class III severe obesity is unique compared to those with class II. While this may seem intuitive, these differences would not have been fully captured utilizing more traditional BMI severity classifications based on BMI percentile, which would have combined class III and class II. For example, while this study found a significant relationship between BMI95pct and HRQoL, when children were categorized and compared by obesity class, it becomes clear that this relationship is being driven largely by the poor HRQoL being reported by those with class III severe obesity. As such, reporting on psychosocial outcomes of children with obesity, which combines these different class groups, may inadvertently miss or misattribute study findings. Additionally, in alignment with other research on HRQoL in pediatric obesity [22,23,34], this study found a difference in BMI95pct and functioning across racial and ethnic groups. Most notably, not only was BMI95pct greater in Black and African American children compared to others, when evaluated categorically, Black and African American children were more likely than other children to present with Class III obesity. Furthermore, when evaluated across the entire sample, Black and African American children reported poorer HRQoL; however, this result did not survive post hoc analyses, so it cannot be said that the racial and ethnic groups differed from each other within each obesity class.

This study supports the use of sub-classifications of obesity and demonstrates that, considering children with severe obesity, according to BMI95pct and obesity class, better accounts for differences in psychosocial functioning (HRQoL) reported by children in this study. Though previous studies have documented differences in psychosocial functioning in children with obesity (95th–99th percentile) compared to children with overweight (85th–95th percentile) and/or children with severe obesity (i.e., ≥99th BMI percentile) [35,36,37,38], the current study showed that children with class III severe obesity had HRQoL, which differed significantly from those with less severe obesity. This may help inform the prioritization of clinical resources for treatment planning and/or clinic development. This further demonstrates that including children with class III severe obesity in the same group with children with class II severe obesity may overlook the unique experiences of these children and lead to either missed effects or potential overgeneralization of relationships and study effects. Clinically, this study supports practices to assess the psychosocial needs and function of children presenting for weight management, having available staff and resources to address identified psychosocial needs, and being prepared for more significant needs for those children with the most severe obesity.

### Strengths and Limitations

The strengths of the current study include the recruitment of a large, ethnically diverse, clinical sample of children with pediatric obesity compared to similar studies that also collect psychosocial measures. This study is also well-powered as per post hoc power analyses to capture the study effects. This sample also includes a broad age range, and a sample that is well distributed across each obesity class, including a substantial number of children with class III obesity. However, the current study is limited in that it only included a single measure of psychosocial functioning. Though this measure of HRQoL is well established, valid, and reliable, generalizations to specific areas of psychosocial functioning (e.g., anxiety and depression) are limited. This study also does not include other clinical measures of risk, such as cholesterol, blood pressure, and blood glucose. Thus, it is not known how well these differences in psychosocial functioning correlate to differences across other clinical metrics. Additionally, while this study is ethnically diverse, and well distributed for biological sex, it is unknown how results could vary according to identified gender. Furthermore, there are other potentially confounding variables (such as family socioeconomic status) that were not included due to a lack of consistency in data collection between treatment programs and lack of availability for this analysis. Finally, the sample was from a single Midwest metropolitan area and would need to be replicated in other rural or regional metropolitan areas to further validate results.

## 5. Conclusions

The primary goal of the current study was to evaluate whether the classification of severe obesity according to BMI95pct and class of obesity provides a meaningful differentiation of children at the upper extreme of pediatric obesity. This study found that by further analyzing children by severity classification, meaningful differences in reported psychosocial functioning were found. This paper supports the recommendations of recent extant literature to utilize BMI95pct as an alternative metric to BMI percentile or BMIz, and future studies should continue to use BMI95pct to classify and evaluate children with severe obesity. Additionally, the clinical implication supports increased evaluation of the psychological impact of obesity, especially those with class 3 severe obesity. Indeed, additional research is needed to determine other variables that may predict HRQoL and/or are related to obesity class.

## Figures and Tables

**Table 1:** Overall Demographics and Demographics by Obesity Class.

		Overall		Class I (*n* = 153)		Class II (*n* = 160)		Class III (*n* = 152)	
		*N*	*%*	*n*	*%*	*n*	*%*	*n*	*%*
Gender									
	Male	215	46.2	68	44.4	68	42.5	79	52.0
	Female	250	53.8	85	55.6	92	57.5	73	48.0
Ethnicity *									
	White/Caucasian	107	23.2	23	21.2 (29.9)	35	21.9 (32.7)	40	26.7 (37.4)
	Black/African American	148	32.1	28	18.5 (18.9)	50	31.3 (33.8)	70	46.7 (47.3)
	Hispanic/Latino	199	43.2	87	57.6 (43.7)	73	45.6 (36.7)	39	26.0 (19.6)
	Other	7	1.5	4	2.6	2	1.3	1	0.7
		***M***	***SD***	***M***	***SD***	***M***	***SD***	***M***	***SD***
Age		10.84	189	10.57	1.90	10.84	1.77	11.13	1.99
BMI-Z		2.35	0.30	2.02	0.15	2.36	0.10	2.70	0.15
BMI95pct		133.09	22.39	112.26	5.34	128.78	5.48	158.59	19.15

* Parentheses show within group percentages for the three largest race/ethnicity groups. *M* = mean; *SD* = standard deviation; BMI-Z = Body Mass Index Z-score.

**Table 2:** Health-Related Quality of Life and Covariates.

	*M* (*SD*)	*S.E._Mean_*	Md	95% CI	Age		Sex	
HRQoL (*n* = 465)					*r* (*p*)	*B* (S.E.)		*B* (S.E.)
Emotion	69.33 (26.93)	1.25	75	66.88–71.85	−0.19 (<0.001) *	−2.49 (0.64)	−0.22 (<0.001) *	−11.51 (2.41)
Physical	76.54 (21.74)	1.01	80	74.55–78.52	0.04 (0.407)	0.506 (0.53)	−0.03 (0.029)	−4.51 (2.02)
Teasing	76.27 (28.06)	1.30	83.33	73.72–78.82	0.07 (0.137)	1.12 (0.68)	−0.12 (0.009) *	−7.01 (2.59)
Positive Attributes	42.32 (21.30)	0.99	38.89	40.81–44.26	−0.18 (<0.001) *	−2.00 (0.52)	0.04 (0.396)	2.09 (1.96)
Avoidance	87.04 (16.93)	0.78	93.33	85.50–88.58	0.06 (0.185)	0.59 (0.41)	−0.07 (0.119)	−2.57 (1.57)
Total HRQoL	68.25 (14.80)	0.69	71.21	66.90–69.60	−0.09 (0.054)	−0.65 (0.36)	−0.13 (0.004) *	−3.83 (1.37)

*M* = mean; *SD* = standard deviation; *S.E._Mean_* = standard error of the mean; 95% CI = 95th percent confidence interval; *B* = beta weight; *r* = Pearson’s correlation; *r_pb_*_=_ point biserial correlations. * Significant after family-wise error correction.

**Table 3:** HRQoL Differences across Obesity Groups.

		Class I (*n* = 153)		Class II (*n* = 160)		Class III (*n* = 152)		
	*F* (*p*)	*M* (*S.E.*)	95% CI	*M* (*S.E.*)	95% CI	*M* (*S.E.*)	95% CI	*d* *
Emotion ^a,b^	8.47 (<0.001)	71.95 (2.07)	67.90–76.02	73.46 (2.11)	69.49–77.42	62.35 (2.08)	58.26–66.44	0.41
Physical ^a,b^	11.24 (<0.001)	79.32 (1.72)	75.94–82.70	80.27 (1.68)	76.97–83.59	69.82 (1.73)	66.42–73.22	0.44
Teasing ^a,b^	16.61 (<0.001)	82.72 (2.19)	78.42–87.02	79.90 (2.13)	75.71–84.09	65.97 (2.20)	61.65–70.29	0.44
Positive Attributes	0.23 (0.792)	42.72 (1.71)	39.37–46.08	42.84 (1.63)	39.57–46.11	41.35 (1.72)	37.98–44.73	0.11
Avoidance ^a,b^	6.93 (0.001)	87.82 (1.35)	85.17–90.48	90.05 (1.32)	87.45–92.64	83.09 (1.36)	80.42–85.77	0.38
Total HRQoL ^a,b^	13.46 (<0.001)	70.22 (1.16)	67.95–72.50	71.06 (1.13)	68.84–73.28	63.31 (1.17)	61.02–65.59	0.50

*F* = ANCOVA F score; *M* = estimated marginal means, adjusting for age and sex; *S.E.* = standard error; *95% CI* = 95th percent confidence interval ^a^—Significant difference between class 1 and class 3; ^b^—Significant difference between class 2 and class 3. *d* *—Cohen’s d for the difference between class III and class II.

**Table 4:** Total HRQoL Differences across Ethnicity Groups and Obesity Classes.

	Overall Total HRQoL		Class I (*n* = 153)		Class II (*n* = 160)		Class III (*n* = 152)	
	*M* (*S.E.*)	95% CI	*M* (*S.E.*)	95% CI	*M* (*S.E.*)	95% CI	*M* (*S.E.*)	95% CI
White/Caucasian (*n* = 107)	65.80 (1.43)	62.98–68.61	69.42 (2.44)	64.60–74.24	69.26 (2.07)	65.17–73.36	59.54 (2.73)	54.16–64.94
Black/African American (*n* = 148)	68.56 (1.24)	66.12–71.00	72.88 (2.60)	67.74–78.03	73.08 (1.83)	69.46–76.70	64.13 (2.08)	60.02–68.25
Hispanic/Latino (*n* = 199)	69.40 (1.09)	67.27–71.53	69.71 (1.49)	66.77–72.64	70.50 (1.48)	67.8–73.43	66.04 (2.96)	60.16–71.88

## Data Availability

The data that support the findings of this study are available from the corresponding author, [W.R.B.] reasonable request.
